# Quercetin Significantly Inhibits the Metabolism of Caffeine, a Substrate of Cytochrome P450 1A2 Unrelated to *CYP*1*A*2*1*C*  (−2964G>A) and *1*F* (734C>A) Gene Polymorphisms

**DOI:** 10.1155/2014/405071

**Published:** 2014-06-15

**Authors:** Jian Xiao, Wei-Hua Huang, Jing-Bo Peng, Zhi-Rong Tan, Dong-Sheng Ou-Yang, Dong-Li Hu, Wei Zhang, Yao Chen

**Affiliations:** ^1^Department of Clinical Pharmacology, Xiangya Hospital, Central South University, Changsha, Hunan 410008, China; ^2^Department of Pharmacy, Xiangya Hospital, Central South University, Changsha, Hunan 410008, China; ^3^Hunan Key Laboratory of Pharmacogenetics, Institute of Clinical Pharmacology, Central South University, 110 Xiangya Road, Changsha, Hunan 410078, China

## Abstract

*Background*. Quercetin is abundant in plants and human diets. Previous studies indicated that quercetin inhibited the activity of CYP1A2, and the combination of quercetin with the substrates of CYP1A2 might produce herb-drug interactions. This research aims to determine the effects of quercetin and the CYP1A2 gene polymorphisms, namely, CYP1A2*1C  (−2964G>A) and *1F (734C>A), on the metabolism of caffeine. *Method*. The experiment was designed into two treatment phases separated by a 2-week washout period. Six homozygous individuals for the CYP1A2*1C/*1F (GG/AA) genotype and 6 heterozygous individuals for the CYP1A2*1C/*1F (GA/CA) genotype were enrolled in the study. Quercetin capsules (500 mg) were given to each volunteer once daily for 13 consecutive days, and after that, each subject was coadministrated 100 mg caffeine capsules with 500 mg quercetin on the 14th day. Then a series of venous blood samples were collected for HPLC analysis. Correlation was determined between pharmacokinetics of caffeine and paraxanthine with caffeine metabolite ratio. *Results.* Quercetin significantly affected the pharmacokinetics of caffeine and its main metabolite paraxanthine, while no differences were found in the pharmacokinetics of caffeine and paraxanthine between GG/AA and GA/CA genotype groups. *Conclusion. *Quercetin significantly inhibits the caffeine metabolism, which is unrelated to CYP1A2*1C (−2964G>A) and *1F (734C>A) gene polymorphisms.

## 1. Introduction

Quercetin, a member of the flavonoids family, is one of the most prominent dietary antioxidants. It is ubiquitously present in vegetables, fruits, tea, and wine as well as countless food supplements. Quercetin was found to have protective effect against various diseases such as osteoporosis, certain cancers, pulmonary, cardiovascular diseases, and aging [1, 2]. Quercetin is a relatively safe drug and globally recognized as an alternative or complementary medicine. The potential herb-drug interaction (HDI) may be the major concern in the coadministration of quercetin and other medicines [3–6].

Cytochrome P450 superfamily (CYP450s) is generally involved in oxidative, peroxidative, and reductive biotransformation of xenobiotics and endogenous compounds, and HDI is always mediated through the CYP450s. Cytochrome P4501A2 (CYP1A2) is one of the major inducible CYP enzymes, which is an essential phase I enzyme for the activation of the major recognized lung carcinogens including aromatic amines and polycyclic aromatic hydrocarbons [7]. Quercetin could inhibit the activity of CYP1A2 and increase the detoxification of the dietary carcinogen PhIP* in vitro* [8, 9]. Our previous research has demonstrated that quercetin inhibited the function of CYP1A2 in healthy volunteers [10], while whether the CYP1A2 gene polymorphisms would affect the HDI between quercetin and other drugs has aroused our curiosity. The present study aims to determine the effects of quercetin and the CYP1A2 gene polymorphisms, namely, CYP1A2*1C (−2964G>A) and CYP1A2*1F (734C>A), on the metabolism of caffeine.

## 2. Methods

### 2.1. Drugs and Reagents

Quercetin capsules (GNC Quercetin 500, 500 mg capsules) were purchased from General Nutrition Centers, Inc., SC, USA. Caffeine, paraxanthine, and acetaminophen (the internal standard) were obtained from Sigma Chemical Co. (St. Louis, MO, USA). Caffeine capsules (100 mg capsules) were purchased from Xinhua Pharmaceutical Company Limited, Shandong, China. HPLC-grade chloroform, isopropanol, formic acid, acetonitrile, and methanol were purchased from Chemical Reagent Factory of Hunan (Changsha, Hunan, China). Ultrapure water was produced by water purification system (Aquapro. CO., LTD, Shanghai, China).

### 2.2. Subjects

Twelve unrelated healthy adult men (mean ± SD: age 23.6 ± 1.7 years; mass index 24.5 ± 3.8 kg/m^2^) were enrolled in the study from a total of 193 healthy Chinese volunteers who had been screened for CYP1A2 genotypes. The genotyping analyses of CYP1A2*1C (−2964G>A) and CYP1A2*1C/*1F (734C>A) were performed by the PCR-restriction fragment length polymorphism method as described previously [11]. Six homozygous individuals for the CYP1A2*1C/*1F (GG/AA) genotype and 6 heterozygous individuals for the CYP1A2*1C/*1F (GA/CA) genotype were selected randomly. Ethical approval for the study protocol was given by the Xiangya Ethics Committee of Central South University, Changsha, Hunan, China. This clinical trial was registered with the Chinese Clinical Trial Registry (No. ChiCTR-TRC-11001277, http://www.chictr.org/cn/proj/show.aspx?proj=129). All informed consents were signed before the experiment. Participants were healthy with no clinically relevant conditions identified in medical history, physical examinations, electrocardiogram, and routine laboratory tests (blood chemistry, hematology, and urine analysis). All subjects refrained from the use of any prescription or nonprescription medications 2 weeks before and throughout the study. They also abstained from grapefruit juice; apples; onions; wine; herbal dietary supplements; caffeine containing beverages including coffee, green tea, and cola; and chocolate or any other medications 2 weeks before the study and throughout the study. The volunteers were served standard meals and were monitored during the experimental period for the development of any possible adverse effects.

### 2.3. Study Protocol

The study had a randomized design with 2 treatment phases separated by a 2-week washout period. In the first phase, after an overnight fast, all subjects were given a single oral dose of 100 mg caffeine capsules with 150 mL water. Then venous blood samples (5 mL) were collected with EDTA-containing tubes before and at 0.25, 0.5, 0.75, 1, 1.5, 2, 2.5, 3, 4, 6, 8, 12, and 24 h after the caffeine intake. After the washout period, quercetin capsules (500 mg) were given to each volunteer once daily for 13 days at 8:00 AM. On the 14th day, each subject was coadministrated 100 mg caffeine capsules with 500 mg quercetin capsules at 8:00 AM, and venous blood samples (5 mL) were collected with EDTA-containing tubes before and at 0.25, 0.5, 0.75, 1, 1.5, 2, 2.5, 3, 4, 6, 8, 12 h, and 24 h after the caffeine intake. The plasma was separated by centrifugation and immediately stored in polypropylene tubes at −20°C until analysis.

### 2.4. HPLC Method for Caffeine and Paraxanthine

Plasma samples were processed and analyzed with the same method, and the concentration of caffeine and paraxanthine was determined by HPLC based on the reported analytical method with some modifications [12, 13]. Agilent 1100 series HPLC equipment (Agilent technologies Inc., Santa Clara, America) was used and a Hypersil BDS C_18_ column (4.6 mm × 200 mm, 5 *μ*m. Dalian, China) coupled with a phenomenex ODS-C18 guard column (4.0 mm × 3.0 mm, 5 *μ*m. CA, America) was applied to separate the analytes. The detection wave length was set at 280 nm. The column temperature was 25°C. The mobile phase was modified by gradient control. The solvents used for elution were phase A (acetonitrile containing 0.2% formic acid (v/v)) and phase B (10 mM ammonium formate acetic acid containing 0.2% formic acid (v/v), pH 3.5). Typical conditions for elution were 98% B (0–4 min), 98–90% B (4–9 min), 90% B (9–15 min), 90–70% B (15–18 min), 70% B (18–21 min), 70–98% B (21–25 min), and 98% B (25–30 min). The analytical intraday coefficients of variation ranged 2.56%–9.48%. The interday coefficients of variation ranged 5.74%–10.91%. The limit of quantification was 10 nmol/L for each analyte. The standard deviation for quality control samples in all analysis batches was less than 15%, and the results of method validation were specific, precise, and repetitive.

### 2.5. Sample Preparation

Plasma samples were prepared according to the literatures [12, 13]. Samples were rapidly thawed under ambient temperature. Then 1.0 mL aliquots of samples were transferred to 10 mL glass tubes with 50 *μ*L acetaminophen (I.S, 10 *μ*g/mL) and 100 *μ*L 10% formic acid lowering pH to 3.5 and vortex-mixed briefly. Samples were extracted with 5 mL chloroform/isopropanol (9 : 1, v/v). After centrifugation for 10 min at 2600 r/min, 4 mL of organic phase was removed to another batch of glass tubes and evaporated under a gentle stream of nitrogen at 40°C. Finally the samples were reconstituted in 100 *μ*L water/methanol (50 : 50, v/v) with 0.2% of formic acid, and 20 *μ*L aliquots were injected into the analytical column.

### 2.6. Pharmacokinetic Analysis

Caffeine metabolite ratio (MR) calculated by AUC_(0–24 h)_ of paraxanthine/AUC_(0–24 h)_ of caffeine was used as the index for CYP1A2 activity. Plasma concentration-time data of caffeine and paraxanthine were analyzed by noncompartmental pharmacokinetic method using DAS software (Drug and Statistics, Version 2.0, Chinese Pharmacological Society, Beijing, China). The peak plasma concentration (*C*_max⁡_) and time to peak plasma concentration (*T*_max⁡_) were directly obtained from the observed concentration-time data. The area under the plasma concentration-time curve (AUC) from time zero to last measured concentration AUC_(0–24 h)_ was calculated according to the linear trapezoidal rule. The terminal elimination rate constant (*k*) was estimated by linear regression of the terminal portion of the ln (concentration-)time curve, and the elimination half-life (*t*_1/2_) was calculated as 0.693/*k* accordingly.

### 2.7. Statistical Analysis

Statistical analysis was performed with the SPSS software for Windows (Version 11.5, SPSS, Chicago, IL). CYP1A2 activity between different phases or groups was analyzed by paired-samples *t*-test. Pharmacokinetic parameters of AUC_(0–24 h)_, *t*_1/2_, *C*_max⁡_, and *T*_max⁡_ for caffeine and paraxanthine before and after 14-day quercetin treatment were analyzed by paired-samples *t*-test. *P* < 0.05 was considered statistically significant.

## 3. Results

Among the 193 healthy volunteers, 42 of the CYP1A2*1C (−2964A) and 135 of the CYP1A2*1F (734A) allelic variants were screened; the frequency of CYP1A2*1C and CYP1A2*1F was 21.8% and 69.9%, respectively. Finally 6 individuals in each genotype of CYP1A2*1C/*1F GG/AA and GA/CA were enrolled in the clinical trial randomly.

**Figure 1:**
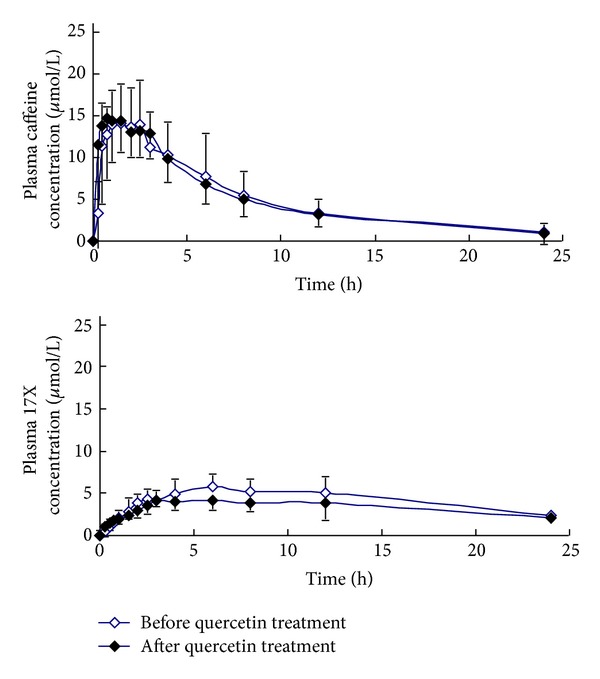
Mean plasma concentration-time profiles of caffeine and paraxanthine (17X) in 6 CYP1A2*1C/*1F GG/AA genotype subjects after a single oral dose of 100 mg caffeine before (hollow) and after (solid) 14-day quercetin treatment.

**Figure 2:**
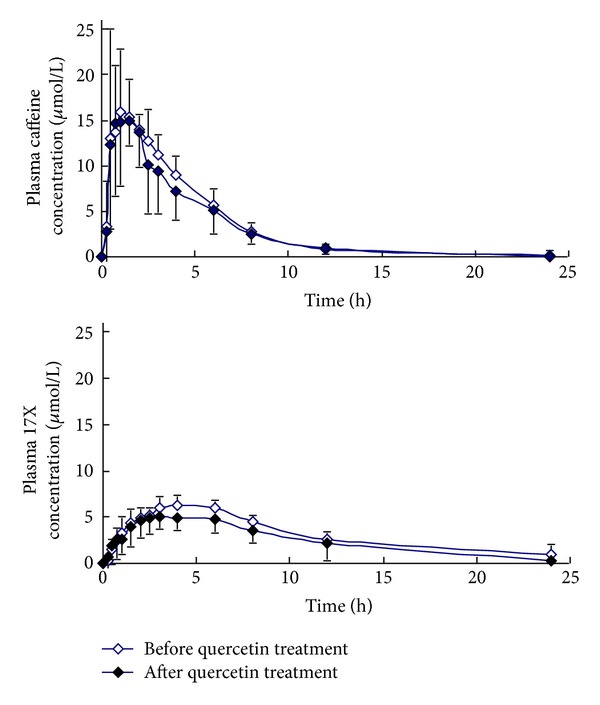
Mean plasma concentration-time profiles of caffeine and paraxanthine (17X) in 6 CYP1A2*1C/*1F GA/CA genotype subjects after a single oral dose of 100 mg caffeine before (hollow) and after (solid) 14-day quercetin treatment.

The *C*_max⁡_ and AUC_(0–24 h)_ values of paraxanthine were decreased by 18.2% [95% CI, 7.5%–29.5%] (*P* = 0.032) and 17.6% [95% CI, 4.9%–29.1%] (*P* = 0.038), respectively, in the GG/AA genotype group during 14-day quercetin treatment phase. Similar results appeared in the GA/CA genotype group: the *C*_max⁡_ and AUC_(0–24 h)_ of paraxanthine were decreased by 17.3% [95% CI, 6.4%–27.4%] (*P* = 0.027) and 16.9% [95% CI, 5.1%–28.2%] (*P* = 0.034), respectively, during 14-day quercetin treatment phase. No difference for *T*_max⁡_ and *t*_1/2_ in the two genotype groups was observed between the two phases. As for caffeine, no significant differences in *C*_max⁡_, AUC_(0–24 h)_, *T*_max⁡_, and *t*_1/2_ were observed during 14-day quercetin treatment or nontreatment phases between the two genotype groups (Figures 1 and 2; Table 1).

**Figure 3:**
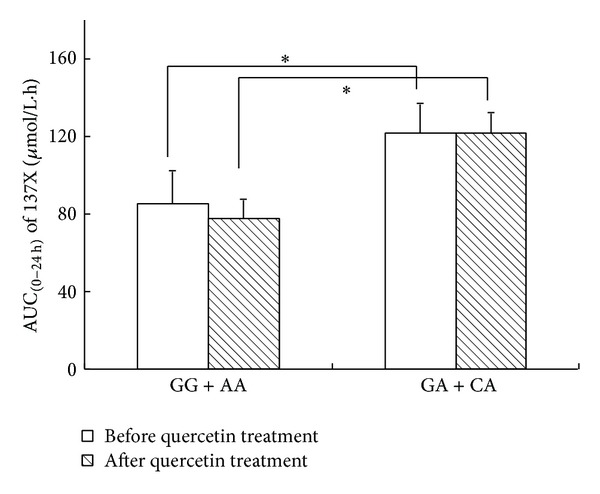
AUC_(0–24 h)_ of caffeine (137X) in subjects after a single oral dose of 100 mg caffeine on day 14 before and after the 14-day quercetin treatment in CYP1A2*1C/*1F GG/AA and GA/CA genotype groups. AUC, area under the plasma concentration-time curve, *P < 0.05.

In both quercetin nontreatment and treatment phases, the AUC_(0–24 h)_ values of caffeine in the GG/AA group were 30.2% [95% CI, 24.5%–46.7%] (*P* = 0.016) and 36.3% [95% CI, 27.8%–48.2%] (*P* = 0.011), lower than those of the GA/CA group, respectively. As for other parameters of caffeine and paraxanthine, no difference was found in any genotype groups or treatment phases (Figure 3; Table 1).

**Figure 4:**
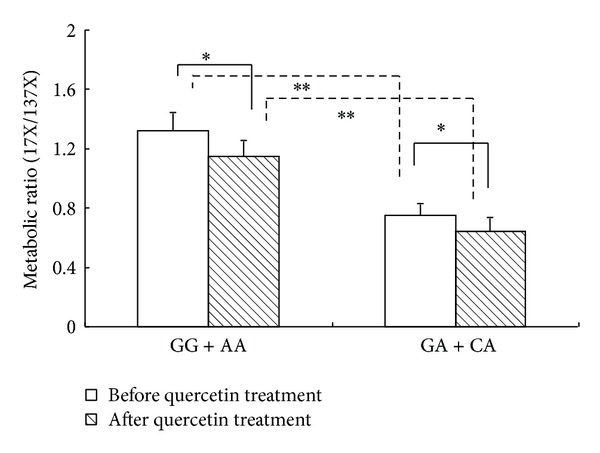
Metabolic ratio of caffeine indicated by AUC_(0–24 h)_ of paraxanthine (17X)/AUC_(0–24 h)_ of caffeine (137X) after a single oral dose of 100 mg caffeine on day 14 before and after 14-day quercetin treatment in CYP1A2*1C/*1F GG/AA and GA/CA genotype groups. AUC, area under the plasma concentration-time curve, *P < 0.05, **P < 0.01.

In both quercetin nontreatment and treatment phases, the MRs of paraxanthine/caffeine in the GG/AA group were 43.2% [95% CI, 29.1%–66.2%] (*P* = 0.005) and 44.3% [95% CI, 30.5%–68.7%] (*P* = 0.002), higher than those of the GA/CA group. Compared with the quercetin nontreatment phase, the MRs were decreased by 12.9% [95% CI, 9.6%–20.3%] (*P* = 0.033) and 14.7% [95% CI, 8.3%–21.4%] (*P* = 0.026), respectively, in the quercetin-treatment phase, while no difference was found in the decrease rate of MRs between the GG/AA and the GA/CA genotype groups (Figure 4; Table 2).

## 4. Discussion

Quercetin is widespread in human diet and globally recognised as an alternative or complementary medicine. Just like its application, the HDI may be the major concern for people [14, 15]. Quercetin could affect the activities of certain CYP450s, for which some researches have provided evidences: the bioavailability of oral doxorubicin, diltiazem, moxidectin, etoposide, and pioglitazone has been significantly increased after coadministration with quercetin, and the underlying mechanism might be through the inhibition of CYP3A [4–6, 16, 17]. In our previous studies, we have demonstrated that 14-day quercetin treatment has significantly decreased the *C*_max⁡_ and AUC of paraxanthine by inhibiting CYP1A2 in healthy volunteers after an oral administration of 100 mg caffeine [10], while whether the CYP1A2 gene polymorphisms affected the HDI between quercetin and caffeine (a substrate of CYP1A2) remained unknown.

CYP1A2 is one of the important enzymes in the oxidative metabolism of exogenous substances. Nearly 84% of caffeine was transformed to paraxanthine by CYP1A2, so caffeine was a typical probe drug in evaluating CYP1A2 activity* in vivo* [10, 11]. CYP1A2*1C (−2964G>A) and CYP1A2*1F (734C>A) are common allelic variants in Chinese population, and the effect of the two mutations on CYP1A2 activity is opposite: mutation of CYP1A2*1C (−2964G>A) could reduce the CYP1A2 enzyme activity induced by smoking, while mutation of CYP1A2*1F (734C>A) could strengthen the induction of smoking on the enzyme activity. CYP1A2*1C/*1F GG/AA and GA/CA genotypes represent the fast metabolizer and the slow metabolizer, respectively [11]. The CYP1A2*1C/*1F AA/CC genotype may represent the lowest enzyme activity group, but due to the low frequency of this genotype, no volunteers were found in screening and we might want to expand 9~10-fold the number of subjects to screen enough AA/CC genotype volunteers, so the lack of AA/CC genotype volunteers might be a shortage in the present research.

In both quercetin nontreatment and treatment phases, the AUC_(0–24 h)_ value of caffeine in the GG/AA genotype group was much lower than that of the GA/CA genotype group, indicating a higher metabolic enzyme activity of CYP1A2 in the GG/AA genotype group than in the GG/AA genotype group, and accordingly a faster elimination of caffeine in the plasma. The MR of paraxanthine/caffeine in the GG/AA genotype group was much higher than that in the GA/CA genotype group in each phase, and the genotype and phenotype were consistent.

The *C*_max⁡_ and AUC_(0–24 h)_ of the main metabolite paraxanthine were decreased by quercetin treatment, which indicated that 14-day quercetin treatment has significantly inhibited the CYP1A2 activity* in vivo*, and consequently suppressed the transformation of caffeine into paraxanthine. Although the MRs were decreased by quercetin treatment in the two genotype groups, we failed to find any significant difference in the inhibition degree on MR between the two genotype groups, neither on the *C*_max⁡_ and AUC_(0–24 h)_ reduction degrees of paraxanthine, indicating that CYP1A2 genotype had similar or no effect on quercetin treatment.

We may come to the conclusion that the 14-day quercetin treatment can significantly inhibit the caffeine metabolism and CYP1A2 activity, which is unrelated to CYP1A2*1C (−2964G>A) and CYP1A2*1F (734C>A) gene polymorphisms. The possible mechanism might be that quercetin inhibits the CYP1A2 activity at the enzyme level, with no effect on the expression at mRNA level. The hypothesis needs to be elucidated by further experiments. Since the CYP1A2 primarily is involved in the metabolism of various drugs such as caffeine, theophylline, phenacetin, clozapine, arachidonic acid, and R-warfarin [18], the potential HDI associated with quercetin should be taken into consideration in clinical practice.

## Figures and Tables

**Table 1:** Pharmacokinetic parameters of caffeine and its metabolite paraxanthine (17X) and the relationship with CYP1A2 genotype in 12 healthy subjects before and after the 14-day quercetin treatment.

Parameters	CYP1A2∗1C/∗1F (GG/AA, *n* = 6)						CYP1A2∗1C/∗1F (GA/CA, *n* = 6)					
	Caffeine			17X			Caffeine			17X		
	Before	After	Ratio	Before	After	Ratio	Before	After	Ratio	Before	After	Ratio
*C*_max⁡_ (*μ*mol/L)	19.8 (13.6, 30.0)	18.3 (14.2, 22.3)	1.2 (0.7, 1.6)	6.6 (5.4, 7.8)	5.3 (4.7, 5.8)	1.2 (1.0, 1.4)	16.6 (12.6, 20.5)	20.4 (12.8, 28.1)	0.9 (0.7, 1.0)	6.6 (5.7, 7.5)	5.8 (4.5, 7.1)	1.2 (0.8, 1.7)
AUC_(0–24 h)_ (*μ*mol/L*·*h)	85.1 (66.8, 103.4)	77.6 (55.4, 99.9)	1.0 (0.8, 1.1)	112.4 (90.8, 134.0)	89.6 (73.8, 105.4)	1.3 (1.1, 1.4)∗	121.9 (81.4, 162.4)	122.0 (93.6, 150.5)	1.2 (0.8, 1.6)	91.5 (77.8, 105.3)	77.9 (61.0, 94.7)	1.3 (1.1, 1.9)∗
*T*_max⁡_ (h)	1.5 (0.5, 2.0)	1.0 (0.5, 1.5)	NA	9 (2, 12)	6 (3, 12)	NA	1.0 (0.5, 2.5)	1.5 (0.9, 3.0)	NA	5.0 (3.0, 6.0)	6.0 (2.0, 10)	NA
*t*_1/2_ (h)	4.5 (3.4, 6.7)	4.5 (3.4, 6.6)	1.2 (0.7, 1.7)	6.6 (4.9, 8.4)	8.0 (6.7, 9.2)	0.8 (0.7, 1.0)	4.1 (3.6, 4.5)	4.1 (3.5, 4.7)	1.1 (1.0, 1.3)	4.9 (4.1, 5.7)	5.4 (4.8, 5.9)	0.9 (0.8, 1.1)

*C*_max⁡_, maximum concentration; *T*_max⁡_, time to maximum concentration; AUC_(0–24)_, area under concentration-time curve extrapolated to 24 h; NA, not applicable; CI, confidence interval. The 90% CI of the ratio of logarithmically transformed parameters was calculated as before quercetin versus after quercetin treatment. AUC_(0–24 h)_ value of caffeine in the CYP1A2∗1C/∗1F (GG/AA) group was much lower than in the CYP1A2∗1C/∗1F (GA/CA) group. *P < 0.05 for comparison for 137 and 17X groups between the phases before and after quercetin treatment.

**Table 2:** CYP1A2 activities (MR) between different CYP1A2 genotypes before and after the 14-day quercetin treatment. Data are expressed as mean ± SD.

Group	MR (before quercetin treatment)	MR (after quercetin treatment)
CYP1A2∗1C/∗1F (GG/AA)	1.32 ± 0.13	1.15 ± 0.11∗
CYP1A2∗1C/∗1F (GA/CA)	0.75 ± 0.08	0.64 ± 0.10∗

*stands for *P* < 0.05; difference in CYP1A2 activities (MR) between the two CYP1A2 genotypes before and after the 14-day quercetin treatment is significant.
